# Pathophysiologic similarities between autism spectrum disorder and Alzheimer's disease: therapeutic possibilities

**DOI:** 10.3389/fnins.2025.1737007

**Published:** 2026-01-20

**Authors:** William Thomas Phillips, Alison P. Sheesley, Joyce Gensberg Schwartz

**Affiliations:** 1 Department of Radiology, UT Health San Antonio, San Antonio, TX, United States; 2 Department of Applied Psychology and Counselor Education, University of Northern Colorado, Greeley, CO, United States; 3 Department of Pathology, Methodist Hospital, San Antonio, TX, United States

**Keywords:** Alzheimer's disease, amyloid, autism pathophysiology, autism spectrum disorder, CSF drainage, glymphatics, nasal turbinates, parasympathetic activity

## Abstract

The comparison of autism spectrum disorder (ASD) and Alzheimer's disease (AD) through shared pathophysiologic features offers intriguing insights into the similarities between the two disease states. The authors suggest diminished cerebrospinal fluid (CSF) drainage through the lymphatic system, perivascular system, and nasal turbinates may occur in ASD and AD, and be an important contributing factor in the occurrence of both disorders. Obstruction of the CSF's normal nasal lymphatic drainage results in abnormal processing of the waste proteins tau and amyloid in the brain in both of these disease states. Reproducible research has shown that ASD and AD patients, when compared to normal controls, exhibit increased extra-axial CSF, enlarged perivascular spaces, magnetic resonance imaging evidence of glymphatic dysfunction, and olfactory dysfunction. Some comparisons between the two disease states are robust while others remain speculative. However, the recognition of overlapping pathophysiologic and genetic features between the two disease states not only furthers understanding of these complex conditions, but could also pave the way for novel therapeutic avenues. The goal of this article is to demonstrate the empirically known similarities between ASD and AD and to stimulate research investigating CSF lymphatic drainage through the nasal turbinates. The authors suggest various ways to confirm their findings and provide suggestions for new therapeutic approaches for these disease states aimed at increasing the movement of CSF originating in the brain through the glymphatic system to meningeal and nasal turbinate lymphatics.

## 1 Introduction

Over the past few decades, in many regions of the world, there has been a steady increase in the incidence of both autism spectrum disorder (ASD) and Alzheimer's disease (AD) (Li et al., 2022b; Solmi et al., 2022; Hirota and King, 2023; Collaborators, 2024). An ongoing discourse persists regarding whether the apparent rise in ASD is attributable to increased exposure to risk factors or is a result of heightened social awareness of not only parents and guardians of children and adults with ASD, but also the awareness of medical staff, enhanced availability of diagnosis, and greater accuracy of diagnostic criteria (Taylor et al., 2020; Solmi et al., 2022). Recent U.S. data from 2022 indicate that ASD may affect 1 in 54 children, and this rate is higher than prior 2014 estimates (Baio et al., 2018; Maenner et al., 2023).

The global incidence of AD is also rising, driven primarily by the aging of the population, with an estimated increased incidence from 1991 to 2019 of 147.9% (Li et al., 2022b; Alzheimers Dement, 2022). The global prevalence of AD is approximately 55 million (Li et al., 2022b).

The healthcare system experiences elevated demand across primary care, mental health, emergency services, and specialty services, as autistic children and adults have higher rates of service use and commonly co-occurring conditions [e.g., intellectual disability, anxiety, depression, attention deficit hyperactivity disorder (ADHD)]. A *JAMA Pediatrics* investigation published approximately 10 years ago found that lifetime societal costs per individual with ASD were roughly $1.4 million (without intellectual disability) to $2.4 million (with intellectual disability), reflecting special education, counseling and therapy services, long-term care, and caregiver time costs (Buescher et al., 2014). Recent years have seen a shift toward neurodiversity-affirming, person-first, play-based treatments such as AutPlay^®^ Therapy, which emphasize regulation and connection rather than behavioral compliance (Chapman and Botha, 2023; Grant, 2023). But still, current therapies only serve to help manage symptoms of ASD that are interfering with social and emotional functioning, and while there is much speculation regarding the causes of ASD (Ahlqvist et al., 2024), only a minimal number of reproducible findings are reported about the causes or prevention of ASD.

Despite extensive research, potential etiological mechanisms of ASD remain unknown. Similarly, in recent years, scientists have made tremendous progress in understanding AD; however, its mechanism of development is not yet fully understood.

In this article, the authors propose a novel contributing factor for ASD based on their review of nuclear medicine scans in which they observed a pattern of significantly increased nasal turbinate vasodilation and blood pooling in patients with high-risk factors for Alzheimer's disease and vascular dementia.

In October 2024, the authors published an article in *Frontiers in Aging Neuroscience* (Phillips and Schwartz, 2024) which described significantly increased nasal turbinate vasodilatation and congestion with resultant nasal lymphatic obstruction of cerebrospinal fluid (CSF) drainage in patients with increased risk factors for AD. The authors propose the same pathophysiology may be common in individuals with ASD.

A review of the literature found many of the signs and symptoms observed in AD to be common to ASD. These similarities include an increased build-up of beta amyloid in the brain, increased extra-axial CSF, enlarged perivascular spaces (EPVS), olfactory dysfunction, hypertension, increased body mass index (BMI), hyperlipidemia, and sleep disorders.

The unique similarities between the two disease states suggest at least some common mechanisms in their development. This article will describe the shared signs and symptoms and provide suggestions for possible new diagnostic treatment modalities.

## 2 Risk factors associated with ASD and AD

The evolving understanding of autism is challenging the traditional view of it as a static, inherited neurodevelopmental condition. Emerging research suggests a complex interplay between genetic susceptibility and environmental exposures, contributing to metabolic and immune anomalies across multiple organ systems, including the brain (Janicki et al., 2025). ASD and AD share some genetic risk factors, exhibit overlap in some pathophysiological mechanisms, and experience some of the same environmental exposures. Families that have members with AD are more likely to have children with ASD (Chang et al., 2025). Autistic adults under the age of 65 are approximately 2.6 times more likely to be diagnosed with younger-onset dementia compared to the general population (Vivanti et al., 2021).

ASD is a neurodevelopmental disorder influenced by a complex interplay of genetic and environmental factors. Recent studies show that adults with ASD are 2.5 times more likely to develop AD than age-matched controls without ASD. In 2024, Lee and Seong performed genome-wide association studies and Mendelian randomization analysis and found synaptic dysfunction in both ASD and AD (Lee and Seong, 2024).

There are documented potentially modifiable risk factors associated with ASD which include a maternal age of 35 years or over [relative risk (RR) 1.31, 95% CI 1.18–1.45], maternal chronic hypertension [odds ratio (OR) 1.48, 95% CI 1.29–1.70], maternal gestational hypertension (OR 1.37, 95% CI 1.21–1.54), maternal overweight before or during pregnancy (RR 1.28,1.95% CI 19–1.36), and pre-eclampsia (RR 1.32, 95% CI 1.20–1.45; Kim et al., 2019; Rivell and Mattson, 2019; Chieh et al., 2021; Katz et al., 2021).

The most common genetic risk factor for the development of AD is the APOE-ε4 gene. And, similar to ASD, modifiable risk factors appear to play a substantial role in AD, accounting for 40% of dementia cases (Reuben et al., 2024). High body mass index (BMI), high fasting plasma glucose, smoking, and obesity are consistently identified as major contributors to the disease burden of AD (Yu et al., 2025).

## 3 The glymphatic system and discovery of meningeal lymphatics

An important understanding of the unique pathophysiology observed in both ASD and AD involves the glymphatic system. CSF, along with waste matter from the brain, drains through the glymphatic system. Any obstruction would impede the normal flow.

The glymphatic system was discovered in 2012 by researchers at the University of Rochester Medical Center. The discovery was made possible by using advanced imaging techniques to visualize the flow of CSF through the brain. This finding revealed a previously unknown network of channels that play a crucial role in clearing waste products from the brain (Iliff et al., 2012).

The glymphatic system consists of perivascular spaces that permit CSF inflow deep into the neural parenchyma (Aspelund et al., 2015; Louveau et al., 2015; Nedergaard and Goldman, 2020; Hablitz and Nedergaard, 2021). The glymphatic system runs in the same direction as blood flow which is propelled by pulsations from the arterial vascular wall. This system transports protein waste products such as amyloid and tau degradation products from the brain via the perivenous spaces (Nedergaard and Goldman, 2020). The fluid in the perivenous space eventually moves into the subarachnoid space on the surface of the brain where this fluid and any waste material are absorbed into meningeal lymphatic vessels (Aspelund et al., 2015; Louveau et al., 2015). This network of meningeal lymphatics serves the same purpose as classical lymphatic drainage and is essential for maintaining neurophysiological homeostasis. The fluid in the meningeal lymphatics is then transported out of the brain and moves through nasal turbinate lymphatics and then into cervical lymphatics. Although the precise anatomic pathway taken by this CSF/lymphatic fluid out of the cranial cavity remains to be clearly defined, the greatest evidence supports its movement along the cranial and spinal nerves, with the olfactory nerve believed to be the most predominant, and its nerve fibers ending in the nasal turbinates (Johnston, 2003; Nagra et al., 2006). Drainage from these meningeal and cervical lymphatics is relatively fast as tracers injected into the brain or CSF accumulate in the cervical lymph nodes within minutes after injection into the brain or CSF (Plog et al., 2015). The discovery of this glymphatic/lymphatic clearance system has clearly shown that CSF and interstitial fluid are directionally transported within the central nervous system (CNS).

Cerebral spinal fluid, along with waste matter from the brain, drains through the glymphatic system and into traditional lymphatics through the nasal turbinates into the cervical lymph nodes. The authors propose a contributing factor in disease states such as ASD and AD may be dilated, blood-filled vessels within the nasal turbinates causing obstruction of the normal CSF flow through the nasal lymphatics/glymphatic system.

## 4 Clinical implications, mechanistic insights, and research gaps

Glymphatic system dysfunction has distinct clinical implications in ASD and AD, reflecting the underlying disease processes and age groups affected. In AD, impaired glymphatic clearance is closely associated with the accumulation of neurotoxic proteins (Aβ and tau), neuroinflammation, vascular impairment, reactive astrogliosis, microglial activation, and vascular impairment, forming a self-reinforcing cycle that drives neurodegeneration (Kamagata et al., 2022; Hsu et al., 2023; Zhong et al., 2023; Cai et al., 2024; Huang et al., 2024; Zhang et al., 2024; Ding et al., 2025; Zhang et al., 2025). In ASD, glymphatic impairment is associated with altered immune markers and possible immune dysregulation; however, direct evidence for vascular pathology is limited (Smedler et al., 2021; Srivastava and O'Reilly, 2025). The interplay between glymphatic clearance, immune signaling, and vascular integrity is central to CNS health, and its disruption appears to contribute to the pathogenesis of both disorders.

Diffusion tensor imaging-analysis along the perivascular space (DTI-ALPS) is a non-invasive magnetic resonance imaging (MRI) technique used to assess the function of the brain's glymphatic system. Lower DTI-ALPS indices and higher perivascular space (PVS) volumes in AD predict faster rates of amyloid accumulation, brain atrophy, and clinical progression, making glymphatic assessment a valuable tool for early diagnosis and (Li et al., 2022c) monitoring (Huang et al., 2024). This glymphatic failure in AD promotes a vicious cycle of protein accumulation, neuroinflammation, and vascular dysfunction, with genetic factors such as APOE ε4 and behavioral factors such as sleep disturbance modulating risk and progression (Rasmussen et al., 2018; Reeves et al., 2020; Li et al., 2022a; Cai et al., 2024; Hu et al., 2024; Ding et al., 2025; Pinho-Correia et al., 2025).

In ASD, glymphatic impairment is observed early in life and is associated with developmental delay, symptom severity, and deficits in sensory-motor integration (Li et al., 2022c; Garic et al., 2023; Sotgiu et al., 2023, 2025; Tian et al., 2025; Wang et al., 2025; Zhao et al., 2025). The DTI-ALPS index correlates with ASD severity and developmental metrics, and mediation analyses suggests that glymphatic dysfunction partially mediates the relationship between white matter integrity and developmental outcomes (Wang et al., 2025; Zhao et al., 2025). Perivascular space (PVS) enlargement and increased extra axial cerebrospinal fluid (EA-CSF) volume may serve as early neuroimaging biomarkers for ASD diagnosis and stratification, particularly in high-risk infants (Garic et al., 2023). Sleep disturbances, which are common in ASD, may further exacerbate glymphatic dysfunction, although the mechanistic links of sleep disruption are less well studied in ASD than in AD (Shen et al., 2018; Garic et al., 2023).

Despite substantial progress, major research gaps remain. There are no published human studies that have directly compared glymphatic system findings between ASD and AD within the same cohort or using harmonized methodologies (Li et al., 2022c; Garic et al., 2023; Sotgiu et al., 2023; Barlattani et al., 2024; Sacchi et al., 2024; Liu et al., 2025; Sotgiu et al., 2025; Tian et al., 2025; Wang et al., 2025). All available evidence is derived from separate, disease-specific studies with methodological heterogeneity and lack of standardized approaches. Longitudinal data are limited, especially in ASD, and no studies have evaluated glymphatic system response to intervention in humans (Li et al., 2022c; Garic et al., 2023; Sotgiu et al., 2023; Barlattani et al., 2024; Liu et al., 2025; Sotgiu et al., 2025; Tian et al., 2025; Wang et al., 2025; Zhao et al., 2025). The absence of harmonized imaging protocols, biomarker assays, and analytical methods precludes robust cross-disease comparisons and highlights the need for future research employing harmonized protocols and direct comparative design (Van Der Thiel et al., 2023; Barlattani et al., 2024; Liu et al., 2025).

## 5 CSF lymphatic drainage through nasal turbinates

Over the past 20 years, significant evidence has been presented to demonstrate that nasal lymphatics are responsible for significant clearance of CSF from the brain. A major proponent of the importance of CSF movement from the subarachnoid space into the nasal turbinate region was Miles Johnston whose work contradicted the most accepted theory—that the majority of CSF is cleared by the arachnoid granulations (Johnston, 2003; Nagra et al., 2006; Koh et al., 2007). As pointed out by Johnston and Papaiconomou (2002), there has been very limited evidence to support the idea that the arachnoid granulations are the primary site of CSF clearance from the brain. There has, however, been significant research supporting the clearance of CSF through the cribriform plate into the nasal turbinate region. In one study, Johnston's group found that 30 min after injection of radiolabeled human serum albumin into the CSF, the tissue that contained the highest activity was the middle nasal turbinate which had approximately 6 times more activity than the peripheral blood (Nagra et al., 2006). In another study, Boulton, Johnston et al. reported that approximately one-half of a protein tracer was transported from the CSF to the blood via extracranial lymphatic vessels (Boulton et al., 1997). This same group found when CSF transport was blocked through the cribriform plate, resting intracranial pressure doubled from 9.2 cm H_2_O to 18.0 cm H_2_O (Mollanji et al., 2002).

Mehta et al. recently reviewed the brain-nose interface as a potential CSF clearance site in humans, emphasizing the importance of nasal lymphatics in CSF clearance. Their noteworthy publication is titled, “The Brain-Nose Interface: A Potential Cerebrospinal Fluid Clearance Site in Humans,” (Mehta et al., 2021).

Since an original report by Schwalbe in 1869 (Schwalbe, 1869), a large body of work in many different species has indicated a role for lymphatic vessels draining CSF in both cranial and spinal regions. Recently published anatomical and quantitative studies have shown abundant evidence that connections between the CSF and the extracranial lymphatic system represent a significant route for CSF drainage (De Leon et al., 2017; Zhou et al., 2022; Spera et al., 2023; Yoon et al., 2024).

Another recent 2023 study in rats using high-resolution imaging was strongly supportive of lymphatic movement along olfactory nerves. The study concluded that the olfactory nerve pathway into nasal turbinate lymphatics is the major route of CSF clearance from the brain (Spera et al., 2023). In an animal model study by Leeds et al., infusion of Ringer's lactate with blue dye into the cisterna magna to increase the intracranial pressure caused a 3-fold increase in cervical lymph node flow and an increase in blue-colored nasal discharge that appeared 48 min after the beginning of the infusion (Leeds et al., 1989). The nasal discharge increased from negligible, before the cisternal infusion, to 11.4 mL/h following the infusion. These studies provide support for significant clearance of CSF from the region of the brain into nasal and cervical lymphatics.

Ma et al. found that lymphatic vessels were the major outflow pathway of CSF for both large and small molecular tracers in mice. They also found a significant decline in CSF lymphatic outflow in aged compared to young mice suggesting that the lymphatic system may represent a target for age-associated neurological conditions (Ma et al., 2017). In another recent study by Yoon et al., a nasopharyngeal lymphatic plexus was found to be a hub for CSF drainage to the deep cervical lymph nodes. This plexus was suggested as a possible target for the treatment of age-related neurological conditions which are known to be associated with decreased CSF transport to deep cervical lymph nodes (Yoon et al., 2024).

Meningeal lymphatic vessels located along the dural sinuses have been shown to drain into the cervical lymph nodes (Da Mesquita et al., 2018), and are coupled with, and receive drainage from, the recently described glymphatic system within the brain (Ringstad and Eide, 2024) that was first described by Iliff et al. (2012).

An imaging study by De Leon et al. showed tracer activity in the nasal turbinates (De Leon et al., 2017) suggesting CSF movement through the cribriform plate and into the nasal turbinate lymphatics. This study also reported that lateral ventricle and superior nasal turbinate CSF clearance abnormalities were found in AD and that ventricular CSF clearance reductions were associated with increased brain amyloid depositions. Consistent with this finding, decreased CSF clearance and increased brain amyloid have been reported in AD (Li et al., 2022d). These reported changes of CSF clearance abnormalities may, however, be secondary changes, and not the primary cause of AD.

Disruption of CSF flow through the olfactory system has been proposed as a contributor to the AD pathogenesis (Ethell, 2014). A recent MRI tracer imaging study also provides support for nasal lymphatic obstruction causing impaired peri-olfactory CSF clearance through the inferior nasal turbinate. This impaired lymphatic clearance through the nasal turbinate was associated with aging, cognitive decline, and decreased sleep quality (Zhou et al., 2022).

## 6 Dilated nasal turbinates with vascular congestion in patients with risk factors of AD

**Figure 1:**
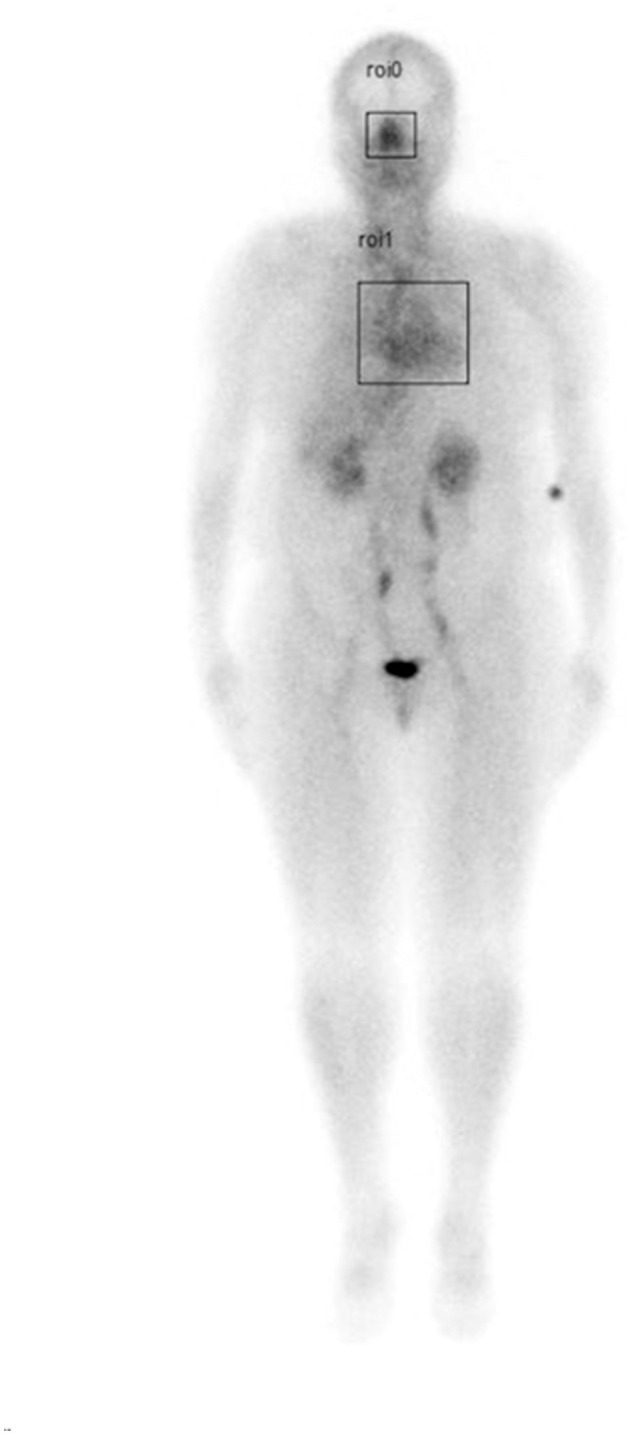
Illustration of a whole-body blood pool scan showing boxed areas (nasal turbinates and cardiac) analyzed for maximum pixel counts to determine nose/heart max ratio. Phillips and Schwartz *Frontiers in Aging Neuroscience*, 2024.

**Figure 2:**
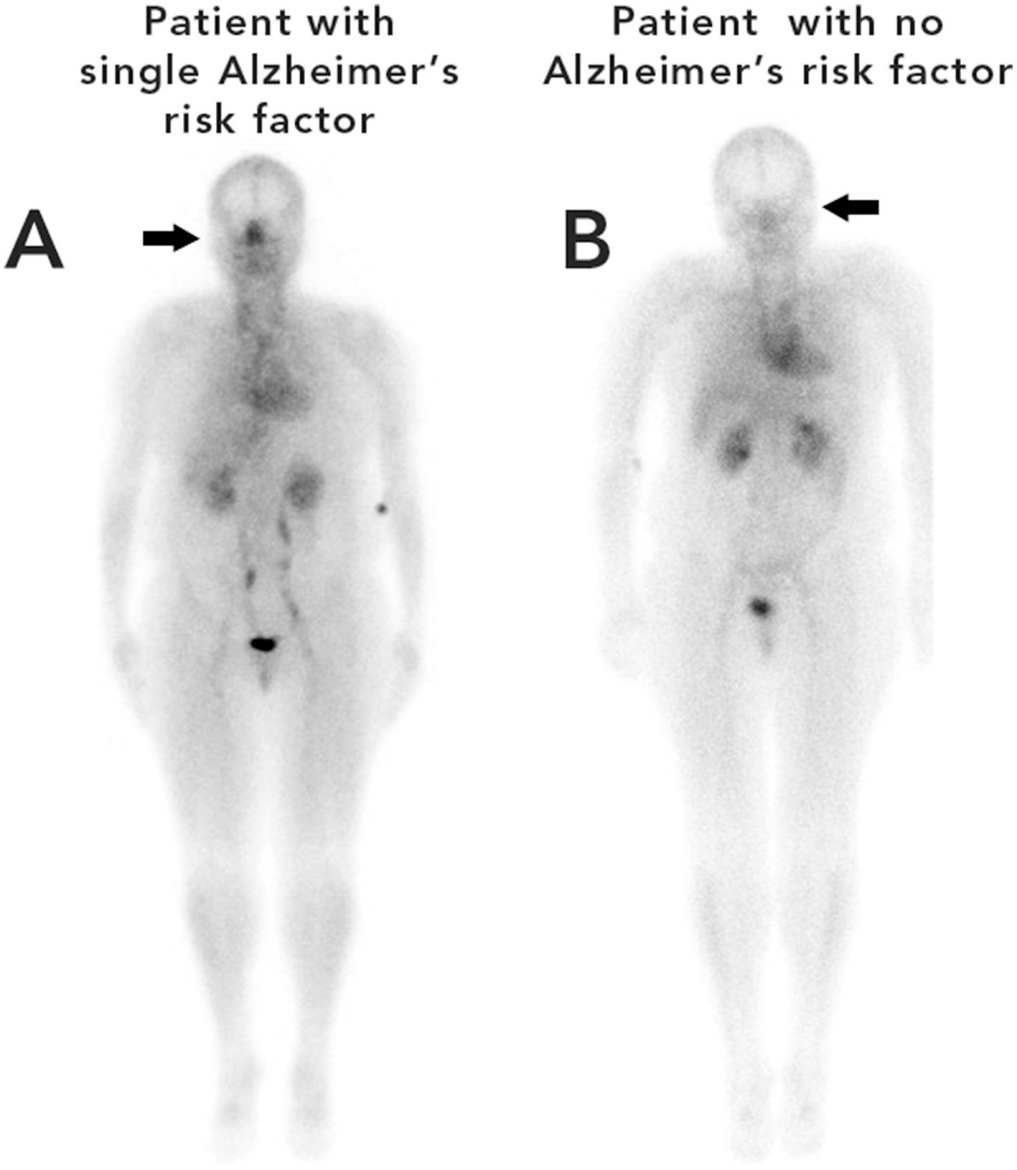
An example of whole-body blood pool scans of two patients with similar BMIs, one (figure **A**) with increased risk factors for AD and the other, (figure **B**) without any risk factors. Phillips and Schwartz *Frontiers in Aging Neuroscience*, 2024. The nasal area in **(A)** shows increased uptake.

The authors believe the same pathophysiologic mechanisms may occur in ASD compared to patients with increased risk factors for AD. In previous studies (Phillips et al., 2022) the authors measured the amount radioactivity (maximum pixel counts) present in the nose and heart regions (creating a nose/heart max ratio) in patients undergoing a whole-body blood pool scan in the department of Radiology at The University of Texas Health Science Center during the years 2017–2020 (Phillips et al., 2022). Many of the patients described in the article had increased risk factors for AD including hypertension, diabetes, sleep apnea, BMI > 25, or elevated glucose/HbA1c values. The patients with increased risk factors for AD had significantly increased nose/heart max ratios (indicating increased nasal vasodilation and blood pooling) on the region of interest analysis of their whole-body blood pool scans as compared to normal controls (Figures 1, 2).

All patients had been referred from the rheumatology clinic at The University of Texas Health Science Center in San Antonio. The study was approved by the Institutional Review Board for a retrospective review (#HSC20200389E). The review took place from May 1, 2017 to May 1, 2020 with patients < 18 years of age and >80 years of age excluded. The rheumatology patients had whole-body blood pool imaging acquired as part of their assessment of joint and other musculoskeletal-related inflammation.

## 7 Imaging acquisition and analysis methodology

All patients were injected with 20–25 mCi of ^99m^Tc-MDP and whole-body blood pool images were obtained beginning at 2–3 min taking 6–7 min to scan from head to feet after injection of the bone avid radiopharmaceutical. This early imaging of the bone avid radiopharmaceutical within the first 10 min after injection is considered to be a marker of the patient's blood pool as the bone avid radiopharmaceutical requires 3 h for bone deposition and clearance of activity from the soft tissues. Images were obtained with a dual-headed gamma camera (GE Infinia Hawkeye 4, Boston, MA) using low energy, high-resolution collimators with an energy window set at 140 keV and with a 20% window moving at a rate of 36 cm per minute.

Table 1 displays the results of Wilcoxson rank-sum tests revealing the nose/heart max ratios were significantly increased in patients with diabetes (*P* = 0.0020), hypertension (*P* = 0.0123), and sleep apnea (*P* = 0.0002) compared to those without these conditions.

**Table 1:** Patients with diabetes, hypertension, or sleep apnea exhibit significantly higher nose/heart max ratios when compared to normal controls.

**Variable**	**No**	**Yes**	***P***
Diabetes	0.86	0.96	0.0020^*^
Hypertension	0.85	0.93	0.0123^*^
Sleep Apnea	0.86	0.99	0.0002^*^

Values are represented as mean (standard deviation). *P* Values depict the Wilcoxon test.

^*^Statistically significant.

Information derived from Phillips et al., *Metab Syndr Relat Disord*, 2022.

Utilizing linear regression analysis, the authors found in their patients, the higher the total number of risk factors for AD, the higher the nose/heart max ratio (Figures 1, 2; Phillips et al., 2022).

## 8 Pathophysiologic similarities between ASD and AD

### 8.1 Cerebrospinal fluid (CSF) dynamics and protein accumulation

Both ASD and AD exhibit disruptions in the normal drainage of CSF (De Leon et al., 2017; Shen, 2018; Zhou et al., 2022; Sotgiu et al., 2023) which may lead to either increased accumulation or altered processing of neurotoxic proteins such as beta-amyloid and tau within the brain. This obstruction can occur due to factors affecting nasal lymphatic drainage, ultimately contributing to neurodegenerative processes.

### 8.2 Decreased brain glucose metabolism

Decreased brain glucose metabolism, also known as brain glucose hypometabolism, refers to a reduction in the brain's ability to utilize glucose as its primary energy source, reflecting impaired glucose uptake, transport, or metabolic processing in brain tissue (Mosconi et al., 2008; Raut et al., 2023).

This phenomenon is particularly significant in neurodegenerative diseases. In AD, glucose hypometabolism appears in preclinical stages—often years before clinical symptoms manifest—and progressively worsens with disease severity (Mosconi et al., 2008; Zhang et al., 2021c). The metabolic deficit may precede and potentially contribute to neurodegeneration rather than simply resulting from neuronal loss (Cunnane et al., 2011; Zhang et al., 2021c).

Both ASD and AD have decreased brain glucose metabolism as measured by 18F-FDG imaging (Keeratitanont et al., 2022). ASD patients have decreased whole-brain glucose metabolism and the left-brain regions are more severely affected than the right-brain regions (Keeratitanont et al., 2022). Alzheimer's patients also have decreased brain glucose metabolism in their parieto-temporal, frontal and posterior cingulate cortices (Mosconi, 2005).

### 8.3 Impaired olfactory function

Olfactory processing issues are frequently seen in both ASD and AD (Bennetto et al., 2007; Crow et al., 2020; Fatuzzo et al., 2023). In AD, early olfactory dysfunction is often a predictor of cognitive decline, while similar deficits in ASD can influence behavior and sensory integration (Bennetto et al., 2007; Dan et al., 2021). Olfactory dysfunction in ASD has been typically interpreted within sensory processing frameworks, and specific glymphatic impairment has not been studied.

### 8.4 Autonomic nervous system abnormalities

There is documented increased **sympathetic activity** of the cardiovascular system in both ASD patients and in patients with risk factors for AD (Bharath et al., 2019; Quarti Trevano et al., 2020; Thapa et al., 2021; Geng et al., 2022; Villar De Araujo et al., 2023). In patients with increased risk factors for AD, our group has observed a simultaneous increase in **parasympathetic activity** of the nasal turbinates and gastrointestinal system (Phillips et al., 1997, 2022). Both ASD and AD have been reported to have an increased number gastrointestinal disorders (Croen et al., 2015; Kuzniar et al., 2024) suggesting the possibility of increased **parasympathetic activity** in these patients as well. While **sympathetic activity** would constrict the nasal vasculature, **parasympathetic activity** would have the opposite effect, and would dilate the vessels. The authors suggest an increased parasympathetic activity in ASD as well as AD is causing vascular dilatation of vessels in the nasal turbinates with blood pooling and resultant vascular congestion similar to the parasympathetic effect on the gastrointestinal system. The authors are aware that autonomic abnormalities in ASD often arise from neurodevelopmental factors and not necessarily from peripheral vasodilatory mechanisms.

## 9 Evidence of glymphatic dysfunction using MRI

Using MRI, both ASD and AD show evidence of glymphatic dysfunction. Both disease states exhibit enlarged perivascular spaces within the brain and extra-axial CSF which is consistent with glymphatic dysfunction and impairment of CSF clearance. Glymphatic dysfunction may have significant effects on brain homeostasis, including the accumulation of neurotoxic substances, altered neuroimmune signaling, and disruption of normal neuronal development (Garic et al., 2023; Frigerio et al., 2025).

### 9.1 Increased extra-axial CSF

**Figure 3:**
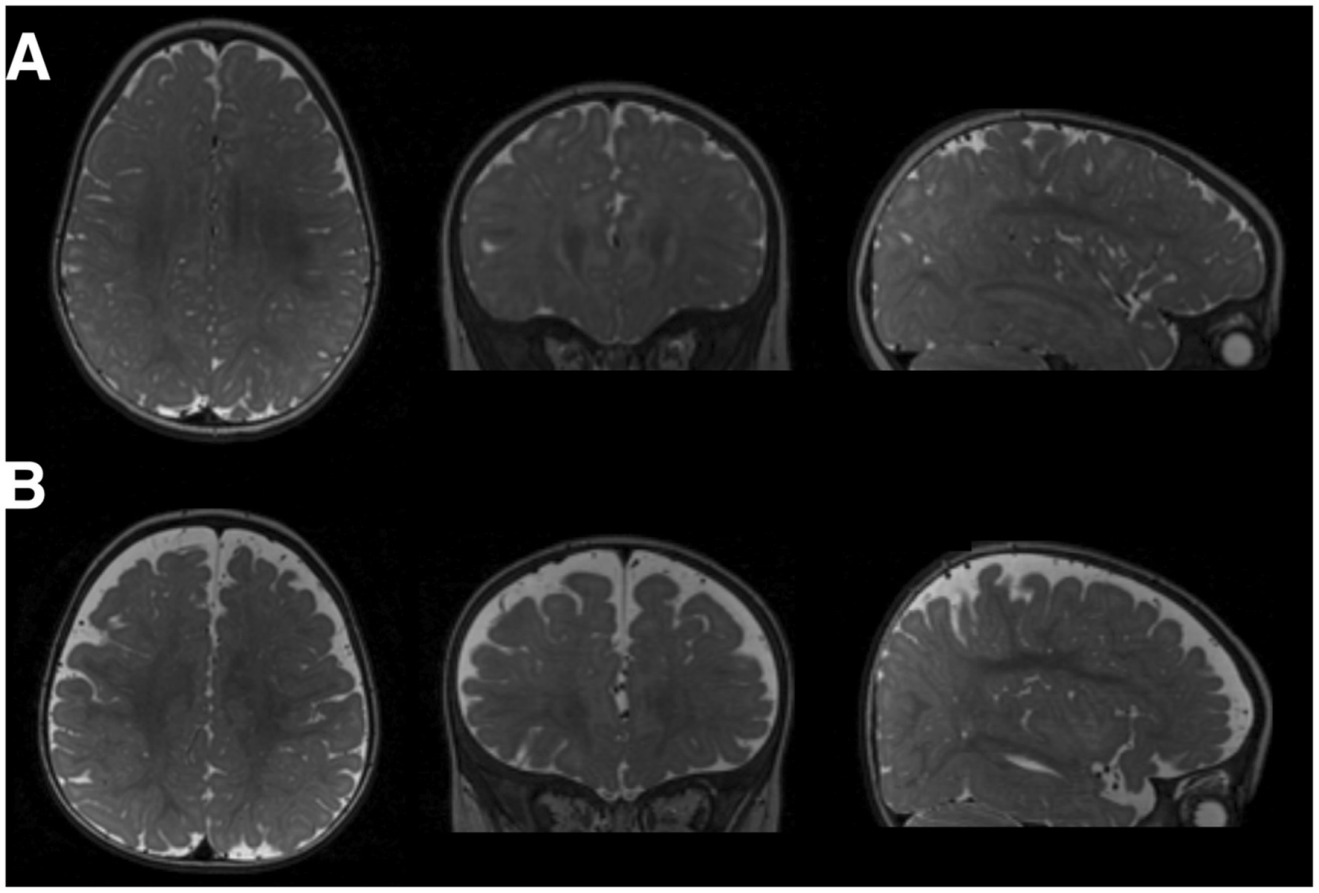
MRI image showing **(A)** normal 6-month-old's brain and **(B)** 6-month-old's brain with extra-axial CSF. Shen, *J Neurodev Disord*, 2018.

Multiple studies have reported that ASD patients have increased extra-axial CSF as compared with normal patients. Figure 3 represents an MRI study of a 6-month-old patient with ASD showing increased extra-axial CSF. The increased extra-axial CSF is considered to be due to decreased CSF clearance rather than increased CSF production (Shen, 2018).

AD patients also have increased extra-axial CSF which may be due to decreased CSF clearance in addition to the well-known brain atrophy occurring in AD (Marino et al., 2019; Zhang et al., 2021a).

### 9.2 Early detection of extra-axial CSF associated with ASD

Quantitative neuroimaging studies over the past decade have established that increased extra-axial cerebrospinal fluid (CSF) volume is significantly more prevalent in infants and toddlers who later develop autism spectrum disorder (ASD) compared to neurotypical controls, although increased extra-axial CSF in ASD is often seen as a part of benign external hydrocephalus in some infants.

The most robust data come from longitudinal MRI studies of high-risk infants, defined as those with an older sibling diagnosed with ASD, when compared to low-risk controls. In a pivotal study by Shen et al. (2013), infants who developed ASD had 20% greater extra-axial fluid than low-risk typical infants at 6–9 months (*P* < 0.01), 33% greater fluid at 12–15 months (*P* < 0.005), and 22% greater fluid at 18–24 months (*P* < 0.005). The increased extra-axial volume of CSF fluid in 6-month-old infants at high risk of developing ASD can be easily observed in Figure 3. Infants with more severe autism symptoms exhibited even greater extra-axial CSF volumes, up to 24% higher at 6 months (*d* = 0.70; Shen et al., 2017). The predictive accuracy of increased extra-axial CSF volume for ASD diagnosis at 24 months was 69%, with a sensitivity of 66% and specificity of 68% (Shen et al., 2017).

The timing of increased extra-axial CSF is notable: it is detectable as early as 6 months of age and persists through at least 24 months, preceding the onset of behavioral symptoms and diagnosis of ASD (Shen et al., 2013, 2017; Ben-Arie et al., 2025). The accumulation of CSF in the subarachnoid space may exert mechanical effects on the developing brain, potentially altering cortical maturation, synaptic pruning, and neuronal connectivity. Quantitative MRI studies have demonstrated that increased extra-axial CSF is associated with larger head circumference and brain volume in ASD, and that the degree of CSF accumulation correlates with symptom severity and lower non-verbal cognitive ability (Shen et al., 2013, 2018; Ben-Arie et al., 2025; Frigerio et al., 2025).

Increased extra-axial CSF volume in ASD may be due to maldeveloped parasagittal dura that potentially disturbs cerebrospinal fluid dynamics as suggested by Agarwal et al. (2024); however, the authors hypothesize that obstruction of the CSF could also be due to lymphatic obstruction at the level of the nasal turbinates.

### 9.3 Enlarged perivascular spaces

Perivascular spaces, previously called Virchow-Robin spaces, are spaces that surround blood vessels in the central nervous system (CNS) (Wardlaw et al., 2020). Perivascular spaces have recently become important for their role in clearance of interstitial fluid and waste from the brain, particularly during sleep. Enlarged perivascular spaces (EPVS) are considered to be due to dysfunction of the glymphatic system (Shulyatnikova and Hayden, 2023).

EPVS can be detected by special imaging sequences of MRI using diffusion tensor imaging (DTI-ALPS) (Cavallari et al., 2023). In recent years, EPVS have been linked to an increased risk of cognitive decline, dementia, stroke, and cerebral small vessel disease (Romero et al., 2022; Yang et al., 2023). One study found that EPVS in the hippocampus was associated with the diagnosis of AD (Gertje et al., 2021). EPVS have been proposed as a potential early biomarker of AD (Lynch et al., 2022) even though the cause of these EPVS is unknown. Various speculations concerning the cause of EPVS have been described including (1) arterial stiffening, (2) protein aggregation, (3) brain atrophy, (4) and destruction of the blood-brain barrier. However, the general consensus is that EPVS are due to impaired glymphatic and meningeal clearance (Shen, 2018; Frigerio et al., 2025). This decreased in glymphatic/meningeal clearance in AD has been proposed by our group to be due to obstruction of lymphatics of the nasal turbinate (Phillips and Schwartz, 2024).

Both ASD and AD share the unique MRI finding of enlarged perivascular spaces (EPVS) (Garic et al., 2023; Menze et al., 2024; Frigerio et al., 2025; Sotgiu et al., 2025). In AD, perivascular space enlargement is more common in males than females with a male to female ratio of 4:1 (Sotgiu et al., 2025). Enlargement of these perivascular spaces has been observed in ASD and is believed to be due to glymphatic dysfunction (Garic et al., 2023).

**Figure 4:**
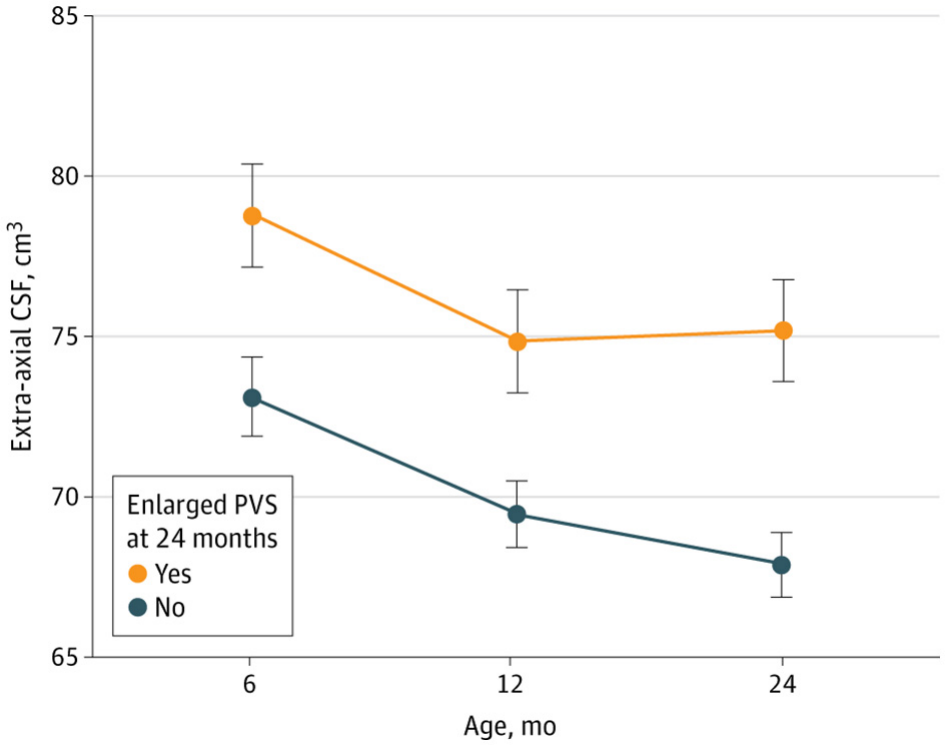
Children with enlarged perivascular spaces at 24 months (EPVS) (orange dots) have significantly greater extra-axial cerebrospinal fluid (CSF) volume compared to children without EPVS at 24 months (blue dots) during the time period from 6 to 24 months of age. Garic et al., *JAMA Netw Open, 2023*.

These EPVS are found in ASD of all ages and in patients diagnosed with AD. In a study of 311 infants by Garic et al., EPVS at 24 months were associated with greater extra-axial CSF volume (*P* = 0.002; Figure 4) and more frequent night wakings in school age children (*P* = 0.006; Garic et al., 2023).

### 9.4 Evidence of glymphatic dysfunction in both ASD and AD using diffusion tensor imaging–analysis

Another method of evaluating glymphatic dysfunction can be evaluated with MRI imaging using diffusion tensor imaging-analysis along the perivascular space (DTI-ALPS) to create a quantitative MRI metric designed to assess water diffusivity along the perivascular spaces in the brain. It has been proposed as a noninvasive surrogate marker of glymphatic system function. The ALPS index is calculated from diffusion tensor imaging (DTI) data by measuring the directionality of water diffusion in specific white matter regions where perivascular spaces run perpendicular to major fiber tracts.

The ALPS index has been validated against direct measures of glymphatic clearance, showing strong correlations (*r* = −0.772 to −0.844) with glymphatic function when measured by intrathecal gadolinium administration. Lower ALPS index values indicate impaired glymphatic clearance function and impaired interstitial fluid dynamics (Zhang et al., 2021b), while higher ALPS index values are interpreted as reflecting a more intact glymphatic function (Taoka et al., 2024; Schirge et al., 2025).

Both ASD and AD patients have been found to have significantly lower DTI-ALPS indices compared to age-matched control subjects (Huang et al., 2024; Li et al., 2022c). The DTI-ALPS index was significantly lower in children with autism when compared with normal controls in the left sided brain index (1.02 ± 0.12 vs. 1.27 ± 0.25, P < 0.001) and in the right sided brain index (1.03 ± 0.12 vs. 1.32 ± 0.20, P < 0.001; Li et al., 2022c). In another recent article, a total of 78 children with ASD and 48 typically developing (TD) children were enrolled. Compared to the TD group, ASD patients showed significantly reduced DTI-ALPS indices in the left hemisphere (DTI-ALPS-L), right hemisphere (DTI-ALPS-R), and whole-brain mean (mean DTI-ALPS; Zhao et al., 2025).

The DTI-ALPS index was also significantly lower in AD dementia when compared with controls (Huang et al., 2024; Schirge et al., 2025). The lower DTI-ALPS index is significantly associated with faster changes in amyloid positron emission tomography (PET) burden, higher risk of amyloid-positive transition and clinical progression, and a faster rate of amyloid and neurodegeneration-related cognitive decline (Hsiao et al., 2023).

## 10 Shared genes between ASD and AD with possible mechanistic connections to the glymphatic system

Genetic overlap between ASD, Alzheimer's disease (AD), and the glymphatic system involves shared risk loci affecting glymphatic clearance function, with common pathways centered on neurovascular integrity, aquaporin regulation, and protein waste accumulation (Table 2).

**Table 2:** Mechanistic connections between ASD and AD involving pathways with potential relationship to the glymphatic system.

**Gene**	**Function**	**Mechanistic connection**	**ASD and/or AD connection**
**ADNP**	Plays a molecular role in microtubule regulation, Tau interaction and axonal transport	Chromatin remodeling and microtubular regulation.	Mutations cause syndromic autism (Helsmoortel et al., 2014) Tauopathy has been shown to be part of the autistic ADNP syndrome (Grigg et al., 2020) Autophagy and ADNP linked as a major target for intervention in brain diseases for AD and ASD (Sragovich et al., 2017) Linked to the glymphatic system in genome-wide association studies (Beer et al., 2024)
**AQP4**	Aquaporin-4 (AQP4) is the principal water channel protein in the central nervous system, where it facilitates bidirectional water transport across cell membranes and plays critical roles in brain water homeostasis, extracellular space regulation, and waste clearance.	Disruption of the glymphatic system leading to increased susceptibility to cerebral edema and neurodegeneration (Ma and Zhang, 2025)	AQP4 altered expression in autistic brains (Fatemi et al., 2008) and in AD (Beer et al., 2024) Altered waste disposal system in AD with a focus on astrocytic AQP4 (Valenza et al., 2019)
**MTHFR**	Deficiency increases phosphorylation of amyloid-B protein precursor potentially promoting pathological amyloidogenic processing (Hoffman et al., 2018)	Disrupted one-carbon metabolism	MTHFR C677T associated with increased risk of late-onset AD (Peng et al., 2015) Gene associated with increased susceptibility to ASD (Li et al., 2020)
**SCN2A, SHANK, PTEN, RELN, FMR1**	Shared genetic risk factors between ASD and AD. Genes converge on synaptic transmission pathways. RELN is particularly important.	Synaptic transmission dysfunction	Its dysregulation through epigenetic mechanisms affects both ASD and AD (Flashner et al., 2013)
**APOE4**	Represents a critical link to glymphatic/meningeal lymphatic dysfunction. May promote premature meningeal lymphatic vessel shrinkage, impairing clearance of amyloid-B, inflammatory mediators, and cellular debris from the brain via the glymphatic-meningeal lymphatic pathway (Mentis et al., 2021)	Impaired waste clearance	Strongest genetic risk factor for late-onset AD and is associated with cerebrovascular dysfunction. Carriers of the APOE4 allele exhibit increased burden of cerebral small vessel disease, including white matter hyperintensities and perivascular space enlargement (Pinheiro et al., 2022). No direct relationship with ASD (Raiford et al., 2004)
**IRS1**	Key docking protein that mediates insulin signaling by transmitting signals from the insulin receptor to downstream pathways that regulate cellular metabolism, growth, and survival (Sesti et al., 2001)	Plays an important role in brain development.	Association between IRS1 Gene Polymorphism and ASD (Park et al., 2016) Genetic variability in IRS1 has been implicated in AD susceptibility (Flores-Dorantes et al., 2020; Fanelli et al., 2022)

Several genes demonstrate direct overlap between ASD and AD pathophysiology. MECP2, ADNP, SCN2A, NLGN, SHANK, PTEN, RELN, and FMR1 are implicated in both disorders (Nadeem et al., 2021). These genes influence neurodevelopmental processes in ASD while also affecting neurodegenerative mechanisms in AD, particularly through their roles in synaptic function, neuronal connectivity, and protein processing (Nadeem et al., 2021).

The APOE ε4 allele represents another genetic link, disrupting meningeal lymphatic function and reducing amyloid beta (Aβ) clearance in AD, while genetic factors affecting neurovascular signaling and blood-brain barrier integrity have been identified in ASD pathogenesis (Ding et al., 2025). Glymphatic disruptions appear to be a shared vulnerability, with genetic variants at the 2p23.3 locus showing concordant effects across both young and aging cohorts, potentially explaining how developmental glymphatic dysfunction in ASD might predispose to accelerated age-related neurodegenerative processes (Huang et al., 2025).

Both Autism Spectrum Disorder and Alzheimer's Disease have strong genetic influences, and recent research points to significant genetic and biological overlaps, suggesting shared pathways like the mTOR signaling system and synaptic regulation which may increase risk for both conditions. Key genes are implicated, and shared genetic factors affecting cellular processes like synaptic plasticity and protein transport highlight the complex link between neurodevelopmental and neurodegenerative disorders.

In the following section, the authors discuss a few of the more frequently cited genes that have been implicated in both ASD and AD. Possible mechanisms of their effect on the glymphatic system will be addressed. At the same time, the authors realize that genetic research in ASD and AD is rapidly evolving with new genetic connections being discovered at an ever-increasing pace.

### 10.1 Activity-dependent neuroprotective protein (ADNP) gene involvement in ASD and AD

Recent whole-exome sequencing studies in autism spectrum disorder (ASD)/intellectual disability (ID) cohorts found activity-dependent neuroprotective protein (ADNP) as one of the most frequently de novo mutated genes, responsible for about 0.2% of ASD cases (Helsmoortel et al., 2014). ADNP is important for brain development and normal function by regulating key genes associated with synaptic transmission including tubulin/microtubules, ion channel and autophagy-controlling genes (Sragovich et al., 2017). Autistic ADNP syndrome has also been shown to have significant tau-related pathology.

Although tau-related pathology is a well-known, critical component of Alzheimer's disease, tauopathy has also been shown to be part of the autistic ADNP syndrome (Grigg et al., 2020). This intersection of ADNP may well represent a critical molecular and pathological intersection between ASD and AD. The relationship between the activity-dependent neuroprotective protein (ADNP) gene and the glymphatic system has been identified through genome-wide association studies, with ADNP emerging as one of the candidate genes linked to glymphatic activity as measured by an ALPS (analysis along the perivascular space) MRI imaging index (Huang et al., 2025).

In a large-scale genome-wide association study of 31,021 individuals, researchers identified 17 unique genome-wide significant loci and 161 candidate genes associated with the ALPS-index, which serves as a marker of brain glymphatic activity (Huang et al., 2025). While the study does not detail the specific mechanism by which ADNP influences glymphatic function, its identification as a candidate gene suggests that ADNP makes a genetic contribution to the regulation of perivascular fluid clearance pathways.

### 10.2 Aquaporin-4 (AQP4)

AQP4 is the major water channel in the brain, predominantly expressed on astrocytic endfeet surrounding blood vessels. It facilitates the glymphatic system—a brain-wide network of perivascular pathways that supports cerebrospinal fluid-interstitial fluid exchange and clearance of interstitial solutes, including amyloid-β (Pedersen et al., 2023). Both deletion and mislocalization of AQP4 significantly impair amyloid-β clearance and promote plaque formation.

Aquaporin-4 (AQP4) dysfunction appears to be implicated in autism pathophysiology, with evidence suggesting that AQP4 deficiency may contribute to autism-like behaviors and that AQP4 expression is altered in autistic brains. Experimental studies demonstrate that chronic inhibition of AQP4 induces autism-like behaviors in rats, including reduced social interaction, increased anxiety, impaired novel object recognition, and decreased locomotor activity—behavioral changes similar to those observed in the valproic acid (VPA) model of autism. This suggests AQP4 deficiency itself may be sufficient to produce autistic phenotypes. Additionally, AQP4 inhibition caused hippocampal water accumulation in control animals comparable to that seen in VPA-exposed offspring (Davoudi et al., 2023).

Genetic variation in AQP4 also influences AD pathology. An AQP4 single-nucleotide polymorphism-based risk score shows significant associations with brain amyloid burden (Beer et al., 2024). The mechanistic link of AQP4 with ASD and AD likely involves disrupted potassium and water homeostasis leading to a significant effect on the glymphatic system.

### 10.3 Methylenetetrahydrofolate reductase (MTHFR) gene

The MTHFR C677T polymorphism is associated with increased risk of late-onset AD (Rai, 2017) and with ASD (Li et al., 2020), particularly in certain populations and specific genetic backgrounds. This relationship appears to be mediated through effects on folate metabolism, elevated homocysteine levels, and downstream impacts on brain structure and amyloid processing. MTHFR is a key enzyme in folate metabolism and one-carbon metabolism, which are critical for DNA methylation and neurodevelopment.

The mechanism likely involves disrupted folate-methionine metabolism. MTHFR deficiency can impair methylation processes and alter neurotransmitter systems. Mouse models show that both maternal and offspring MTHFR genotypes contribute to autistic-like behaviors, with effects on GABAergic and glutamatergic systems. Children with ASD often exhibit metabolic disturbances including elevated homocysteine and reduced methionine, cysteine, and glutathione levels, particularly in those with the C677T polymorphism (Roufael et al., 2023).

While both MTHFR deficiency and glymphatic dysfunction are independently associated with neurodegenerative diseases, particularly Alzheimer's disease, no studies have examined whether MTHFR variants directly affect glymphatic function or whether glymphatic impairment influences folate metabolism. The cerebrovascular deficits caused by MTHFR deficiency could theoretically impact perivascular fluid flow, but this remains unexplored in the medical literature.

## 11 Lifestyle and physiological similarities between ASD and AD

### 11.1 Obesity, maternal diabetes, and increased consumption of energy-dense foods -increased incidence of ASD and AD

In 2019, Rivell and Mattson proposed that the recent increase in the prevalence of ASD was caused by excessive consumption of energy-dense foods, particularly fructose, and consequent obesity and insulin resistance (metabolic syndrome) (Rivell and Mattson, 2019). They also suggested that maternal insulin resistance and obesity may predispose offspring to ASD by mechanisms involving chronic activation of anabolic cellular pathways and a lack of metabolic switching to ketosis resulting in a deficit in GABAergic signaling and neuronal network hyperexcitability. This risk factor may be present even if the mother is within normal weight range, indicating that this relates to a biological vulnerability or predisposition. Other researchers have claimed maternal obesity, maternal diabetes, and gestational diabetes are risk factors for ASD (Katz et al., 2021; Chen et al., 2023). Similarly, the authors also hypothesized in their 2024 article that the rapid increase in worldwide AD was due to the massive increase in consumption of high-energy, high-calorie, highly processed food (Phillips and Schwartz, 2024).

### 11.2 Other shared medical risks between ASD and AD

Shared medical risks between individuals with ASD and AD include a higher risk of having hypertension, hyperlipidemia, and an increased body mass index (BMI) (Croen et al., 2015; Silva et al., 2019; Dhanasekara et al., 2023). ASD and AD patients also have been shown to have decreased heart rate variability (Jensen-Dahm et al., 2015; Bharath et al., 2019; Thapa et al., 2021; Geng et al., 2022) which is consistent with increased sympathetic activity of the cardiovascular system.

### 11.3 Sleep disorders

Sleep is the time of greatest activity of the glymphatic/lymphatic system in clearing brain waste products. Sleep disturbances are prevalent in individuals with ASD and in those with AD (Reynolds and Malow, 2011; Xie et al., 2013; Minakawa et al., 2017; Liguori and Placidi, 2018; Malhotra, 2018; Wu et al., 2019; Zaffanello et al., 2023). Decreased brain waste clearance during sleep can potentially impact cognitive function and overall health. Addressing sleep issues could be crucial in managing symptoms associated with both ASD and AD.

### 11.4 Similar features/parameters between Alzheimer's disease and autism spectrum disorder

Table 3 lists many of the shared clinical and pathophysiologic characteristics that have been documented between autism spectrum disorder and Alzheimer's disease.

**Table 3:** Similar clinical and pathophysiologic characteristics between individuals with Alzheimer's disease and autism spectrum disorder.

**Feature/parameter**	**Alzheimer's disease (AD)**	**Autism spectrum disorder (ASD)**
DTI-ALPS and DKI-ALPS indices	Reduced vs. controls; stage-dependent (e.g., AD: 1.32 ± 0.14, MCI: 1.37 ± 0.13, subjective cognitive decline (SCD): 1.51 ± 0.08; *P* = 0.001; Zhang et al., 2025) ALPS index predicts amyloid PET (Huang et al., 2024)	DKI-ALPS indices were significantly lower in children with ASD than in typically developing children (1.48 ± 0.15), with lower values in the severe autism subgroup (1.32 ± 0.10) than in the mild subgroup (1.40 ± 0.11; Tian et al., 2025)
Perivascular space (PVS) number and volume	Enlarged (Menze et al., 2024); Increased PVS volume (Zhang et al., 2024)	Increased number and size of perivascular spaces correlated with developmental delays (Garic et al., 2023; Frigerio et al., 2025)
Disease correlates	Cognitive decline, disease progression; atrophy, clinical progression (Atri, 2019)	Developmental delay, cognitive impairment, lack of social skills, and stereotyped behavior (Bhat et al., 2014)
Sleep disturbance and glymphatic function	Sleep disturbances common. Glymphatic activity reduced with sleep disturbance; sleep-dependent clearance (Shen, 2018; Garic et al., 2023; Sotgiu et al., 2023)	Sleep disturbance common. May exacerbate glymphatic dysfunction (Reynolds and Malow, 2011; Cortese et al., 2020)
Pathophysiological consequences	Neurodegeneration, protein aggregation, neuroinflammation, vascular impairment (Crews and Masliah, 2010)	Neurodevelopmental impairment, altered immune markers, possible immune dysregulation (Onore et al., 2012; Pretzsch et al., 2024)
Shared genetics between ASD and AD	CR1 (rs670173), CLU (rs7982, BIN1 9rs744373) AD associated gene variants associated with increased risk of ASD (Hoidy et al., 2025)	ADNP gene associated with AD and autism (Ivashko-Pachima et al., 2021)
Shared genetics between ASD and AD related to the glymphatic system	MECP2, ADNP, (Nadeem et al., 2021); APOE ε4 allele (Ding et al., 2025); variants at the 2p23.3 locus (Huang et al., 2025); AQP4 (Valenza et al., 2019)	MECP2, ADNP (Nadeem et al., 2021); APOE ε4 allele (Ding et al., 2025); variants at the 2p23.3 locus (Huang et al., 2025); AQP4 (Fatemi et al., 2008)

## 12 Future confirmatory studies

Studies to confirm the hypothesis of increased parasympathetic activity in ASD causing nasal turbinate vasodilatation and obstruction of normal CSF clearance from the brain could be achieved by examining nasal turbinates. MRI studies of the nasal turbinates could be used to look for evidence of lymphatic obstruction in this region, analogous to enlarged perivascular spaces. Retrospective MRI or CT studies of known ASD patients would enable examination of the nasal turbinates to look for evidence of bilateral vasodilation. Other studies could examine and determine if ASD patients have an abnormal nasal cycle (Kahana-Zweig et al., 2016). An abnormal nasal cycle would suggest decreased CSF clearance through the nasal turbinates. Aging, a risk factor for AD and dementia, has been associated with decreased function of the nasal cycle (Mirza et al., 1997; Williams and Eccles, 2015).

Nuclear blood pool imaging could be performed in patients with ASD to look for increased nasal turbinate vasodilation. These blood pool studies would be similar to our previous studies in patients with risk factors for Alzheimer's disease.

## 13 Diagnostic and therapeutic implications

The proposed common pathophysiology suggests the possibility of utilizing diagnostic tools and therapeutic modalities that could be beneficial for individuals with either ASD or AD. The authors suggest the following.

### 13.1 CSF flow assessment

Techniques to evaluate CSF flow dynamics may help in early diagnosis and monitoring disease progression for both disorders.

### 13.2 Targeted therapies

Interventions focusing on enhancing nasal drainage and CSF circulation could potentially alleviate some symptoms. Both ASD and AD have been proposed to benefit from therapies that enhance glymphatic/lymphatic clearance that would remove waste material from the brain (Shen, 2018; Sun et al., 2018; Nedergaard and Goldman, 2020). Methods of increasing glymphatic/lymphatic clearance could involve insertion of a surgical shunt around the normal lymphatic drainage pathway (Jessup et al., 2025; Plog et al., 2025). This method has already been proposed as a treatment for Alzheimer's disease. Another method would involve increasing the lymphatic flow through the nasal turbinates, which has been proposed by our group as a potential treatment of Alzheimer's disease (Phillips and Schwartz, 2024). This type of therapy could range from pharmacological agents to lifestyle modifications promoting better vascular health.

Studies are currently underway to increase CSF flow as an approach to treating AD. For instance, Jin et al. have shown that massaging of the cervical lymphatics will significantly increase CSF clearance from the brain (Jin et al., 2025). In another approach, restoration of cervical lymphatic flow has been shown by application of a topical prostaglandin PGF2α (Du et al., 2024). Other therapies could be aimed at decreasing the nasal turbinate vasodilation though modification of parasympathetic activity of the nasal turbinates through either pharmacological means (Baraniuk, 1992) or through methodologies focused on nerves that delivery parasympathetic activity to the nasal turbinates (Ho et al., 2017).

## 14 Hypothesis limitations

### 14.1 Empirically supported vs. hypothetical links in the proposed pathway

The proposed pathway of our hypothesis proceeds as follows: nasal turbinate vasodilation and blood pooling → lymphatic CSF obstruction → glymphatic failure → protein (waste product) accumulation → ASD/AD pathology.

There is empirical evidence that strongly supports the last three links in this pathway. Compared to normal controls, patients with either ASD or AD exhibit reproducibly reported magnetic resonance imaging (MRI) evidence of glymphatic dysfunction as supported by increased extra-axial CSF, enlarged perivascular spaces (EPVS) and lower DTI-ALPS indices (Chen et al., 2025; Ding et al., 2025; Frigerio et al., 2025). Empiric evidence also supports significant genetic overlaps between ASD and AD, although the relationship is complex and involves both shared genes and common molecular pathways (Hacohen-Kleiman et al., 2018; Ivashko-Pachima et al., 2021). There is also empiric evidence showing that both ASD and AD patients suffer from sleep disturbances (Reynolds and Malow, 2011; Gottesman et al., 2024) and olfactory dysfunction (Bennetto et al., 2007; Dan et al., 2021; Fatuzzo et al., 2023). The first two links in this pathway, which include nasal turbinate vasodilation and lymphatic CSF obstruction of nasal turbinate lymphatics, are more speculative, and are part of our hypothesis.

There is evidence, much of which is new, to support the normal movement of significant amounts of CSF through the cribriform plate and the nasal turbinates (De Leon et al., 2017; Chae et al., 2024). The percent of CSF clearance through the cribriform plate has been shown to be more than 50% in animal models (Johnston et al., 2004; Nagra et al., 2006; Algin et al., 2025), although, to date, this high percentage has not been confirmed in human studies. As far as nasal turbinate vasodilation, there is currently no evidence to support nasal turbinate vasodilation in ASD; however, there is evidence, recently reported by our group, to support nasal turbinate vasodilation occurring in patients with risk factors for Alzheimer's disease (Phillips et al., 2022).

It is the goal of this article to demonstrate the empirically known similarities between ASD and AD and to stimulate research investigating CSF lymphatic drainage through the nasal turbinates. Further research in this area could show that the nasal turbinates lymphatics are indeed a site of CSF obstruction. If nasal turbinate obstruction is proven, further research regarding therapies targeted at increasing CSF flow through the nasal turbinates with the goal of clearing waste products from the brain and improving ASD and AD symptoms may prove to be useful.

### 14.2 Remaining questions

There remain several questions, however, that this study could not address. *Is there nasal vasodilation with blood pooling obstructing the normal flow of CSF through the nasal lymphatics in individuals with ASD? Does this impair their sleep cycle?*

The authors' hypothesis currently relies on conceptual alignment rather than demonstrated causality. The potential mechanism described by the authors is only one of many plausible mechanisms, but it is one that has emerged as a tractable hypothesis to test experimentally, by virtue of the methods that have been established through the recent discovery of the glymphatic system of the brain. It is unclear whether obstruction of normal lymphatic flow is related to one etiology of ASD, or is an indication of a different underlying process that produces ASD. Given the heterogeneity of autism, it is unlikely that increased nasal lymphatic obstruction is present in all individuals with ASD. However, MRI findings consistent with glymphatic obstruction may delineate one biological subtype of autism spectrum disorder that potentially shares a common underlying biology with Alzheimer's disease. Considering that a subset of ASD patients has MRI findings consistent with glymphatic obstruction, this subset of ASD could benefit from therapies targeted at increasing CSF lymphatic flow through the nasal turbinates.

## 15 Conclusion

The authors have described many clinical and physiologic similarities between ASD and AD. Some comparisons are robust while others remain speculative. However, the recognition of overlapping pathophysiologic features between the two disease states not only furthers understanding of these complex conditions but could also pave the way for novel therapeutic avenues. By concentrating on the mechanisms of waste clearance, inflammation, and metabolic health, researchers may develop integrated treatment strategies that address both disorders simultaneously, ultimately improving the quality of life for affected individuals. Such explorations also underscore the importance of interdisciplinary research in uncovering the intricate relationships between different neurodevelopmental and neurodegenerative diseases. The authors hope their hypothesis regarding the potential of nasal turbinate vasodilation as a contributing factor or therapeutic target in patients with ASD will encourage further research in this area.

## Data Availability

The original contributions presented in the study are included in the article/supplementary material, further inquiries can be directed to the corresponding author/s.
